# Supplementary material for: Synthesis, Self-Assembly in Crystalline Phase and Anti-Tumor Activity of 2-(2-/4-Hydroxybenzylidene)thiazolo[3,2-a]pyrimidines
Source: Molecules. 2022 Nov 10;27(22):7747. doi: 10.3390/molecules27227747 (PMC9693259; doi:10.3390/molecules27227747)

Supplementary Materials for:

Synthesis, self-assembly in crystalline phase and antitumor activity of 2-(2- / 4-hydroxybenzylidene)thiazolo[3,2-*a*]pyrimidines

Artem S. Agarkov [1], Anna A. Nefedova [1], Elina R. Gabitova [2], Alexander S. Ovsyannikov [1], Syumbelya K. Amerhanova [1], Anna P. Lyubina [1], Alexandra D. Voloshina [1], Pavel V. Dorovatovskii [3], Igor A. Litvinov [1], Svetlana E. Solovieva [1] and Igor S. Antipin [1,*]

Arbuzov Institute of Organic and Physical Chemistry, FRC Kazan Scientific Center, Russian Academy of Sciences, Arbuzova 8, 420088 Kazan, Russia;

2 Kazan Federal University, Kremlevskaya 18, 420008 Kazan, Russia;

3 National Research Center “Kurchatov Institute”, Akademika Kurchatova 1, 123182 Moscow, Russia;

* Correspondence: iantipin54@yandex.ru

Figure S1. ^{1}H NMR spectrum of compound **10** (DMSO-d_6, 400 MHz, 25^{0}C).

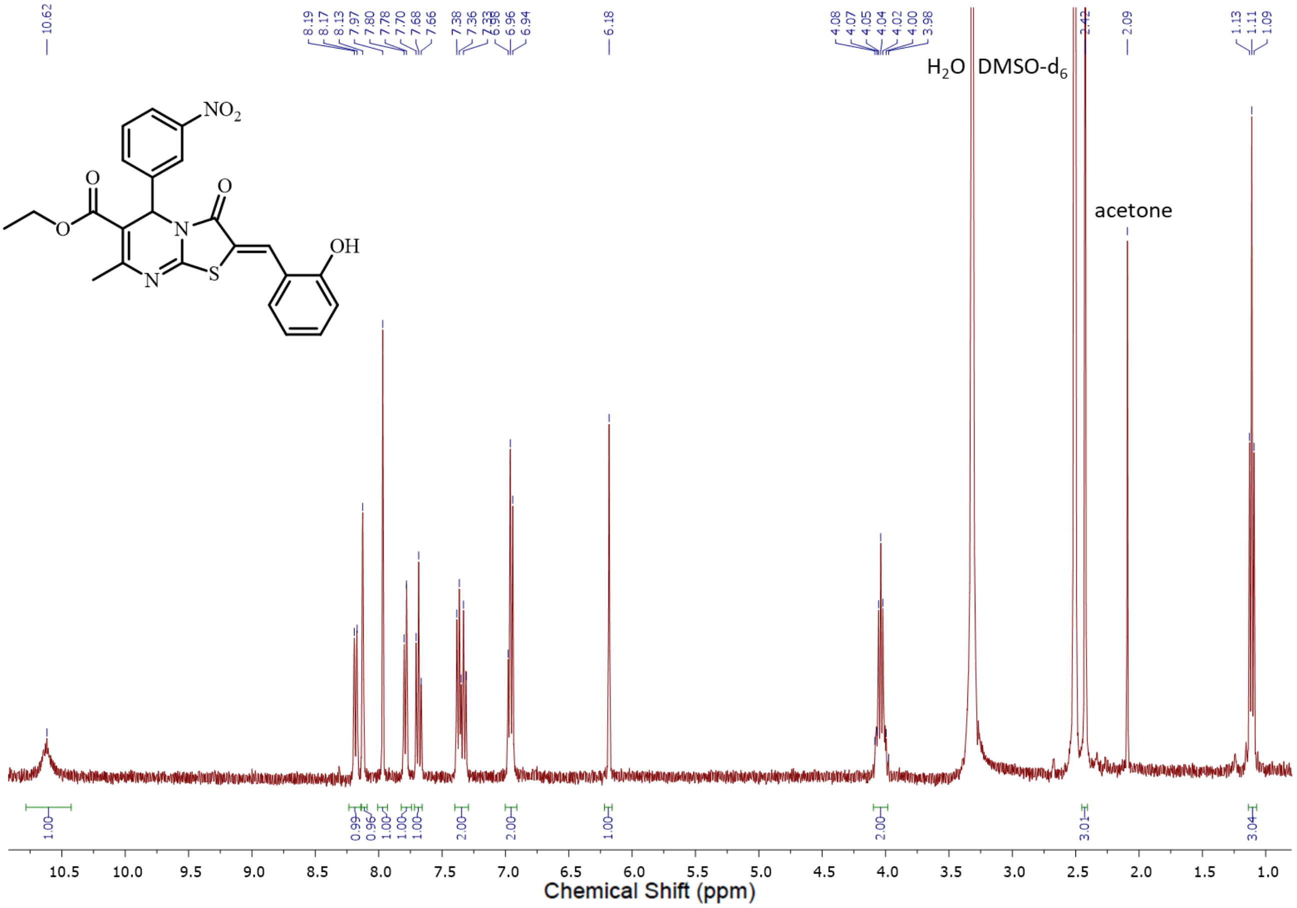

Figure S2. ^{13}C NMR spectrum of compound **10** (DMSO-d$_6$, 100 MHz, 25^{0}C).

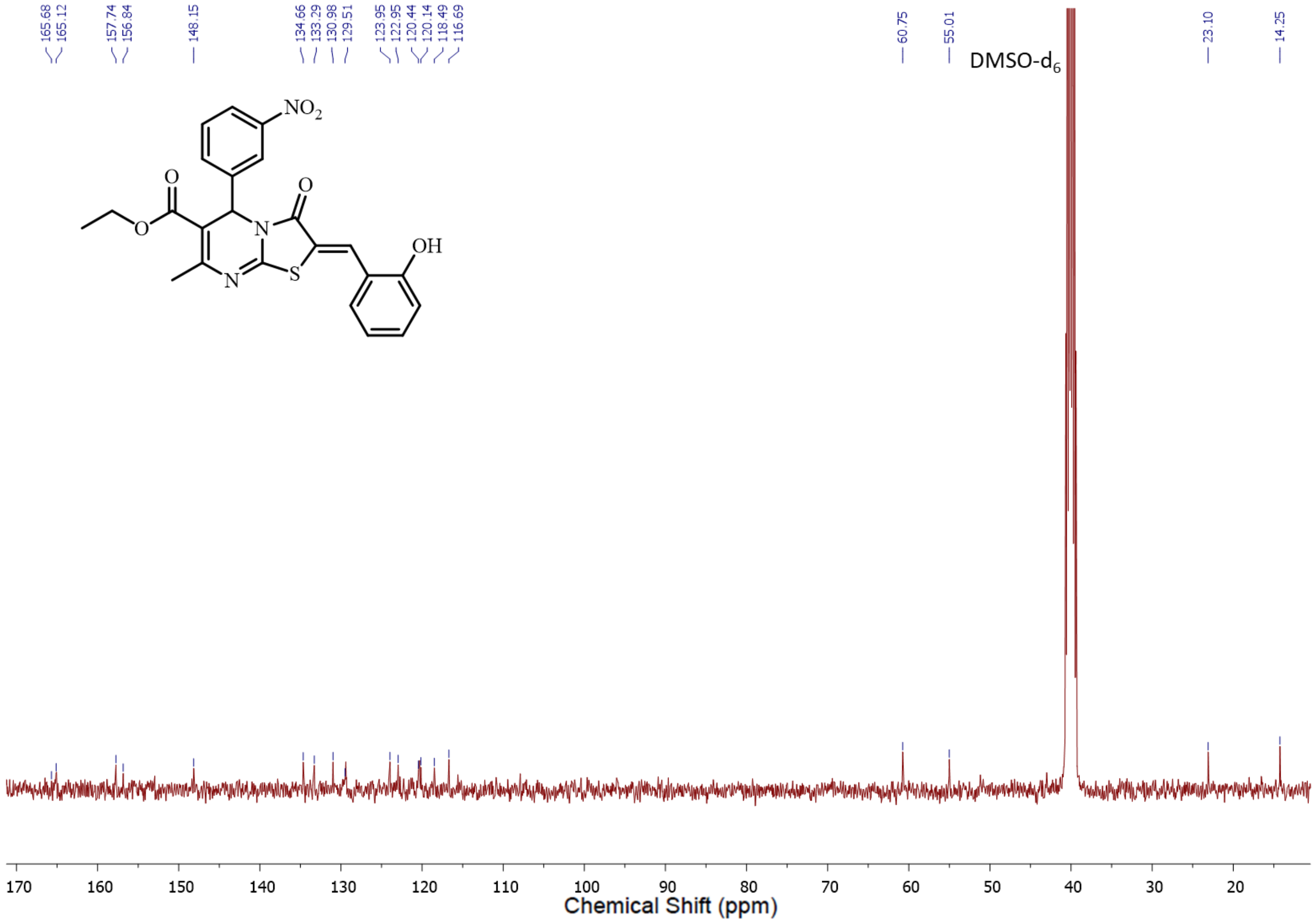

Figure S3. ESI MS spectrum of compound **10** (Ion Polarity: Positive).

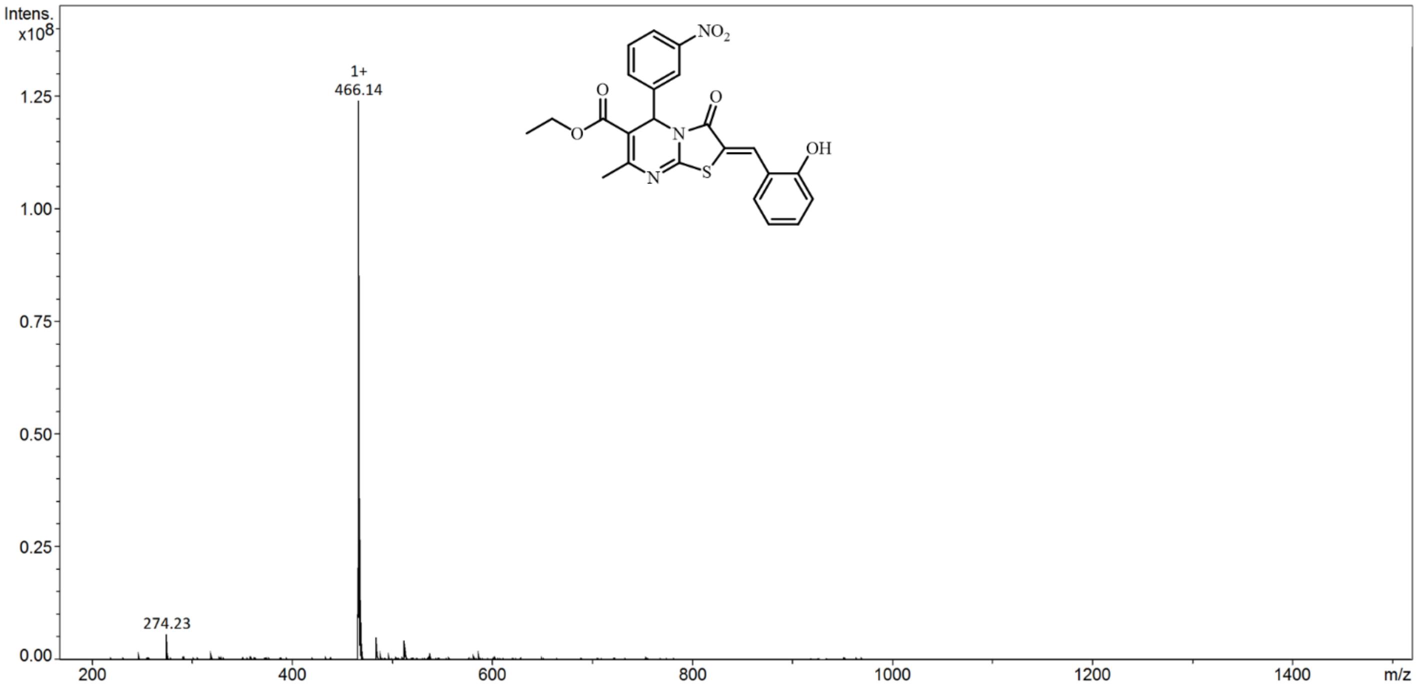

Figure S4. IR spectrum of compound **10** (KBr tablet).

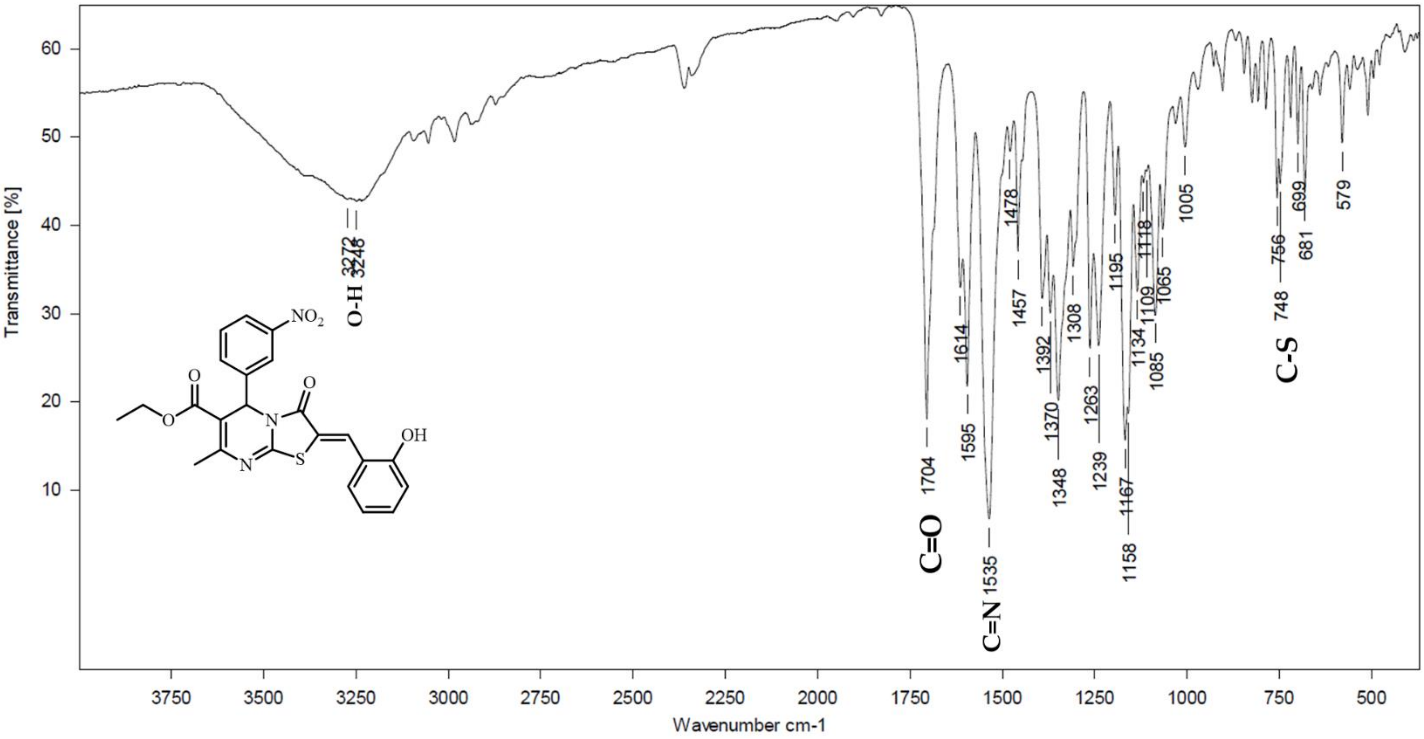

Figure S5. ^{1}H NMR spectrum of compound **11** (DMSO-d6, 400 MHz, 25⁰C).

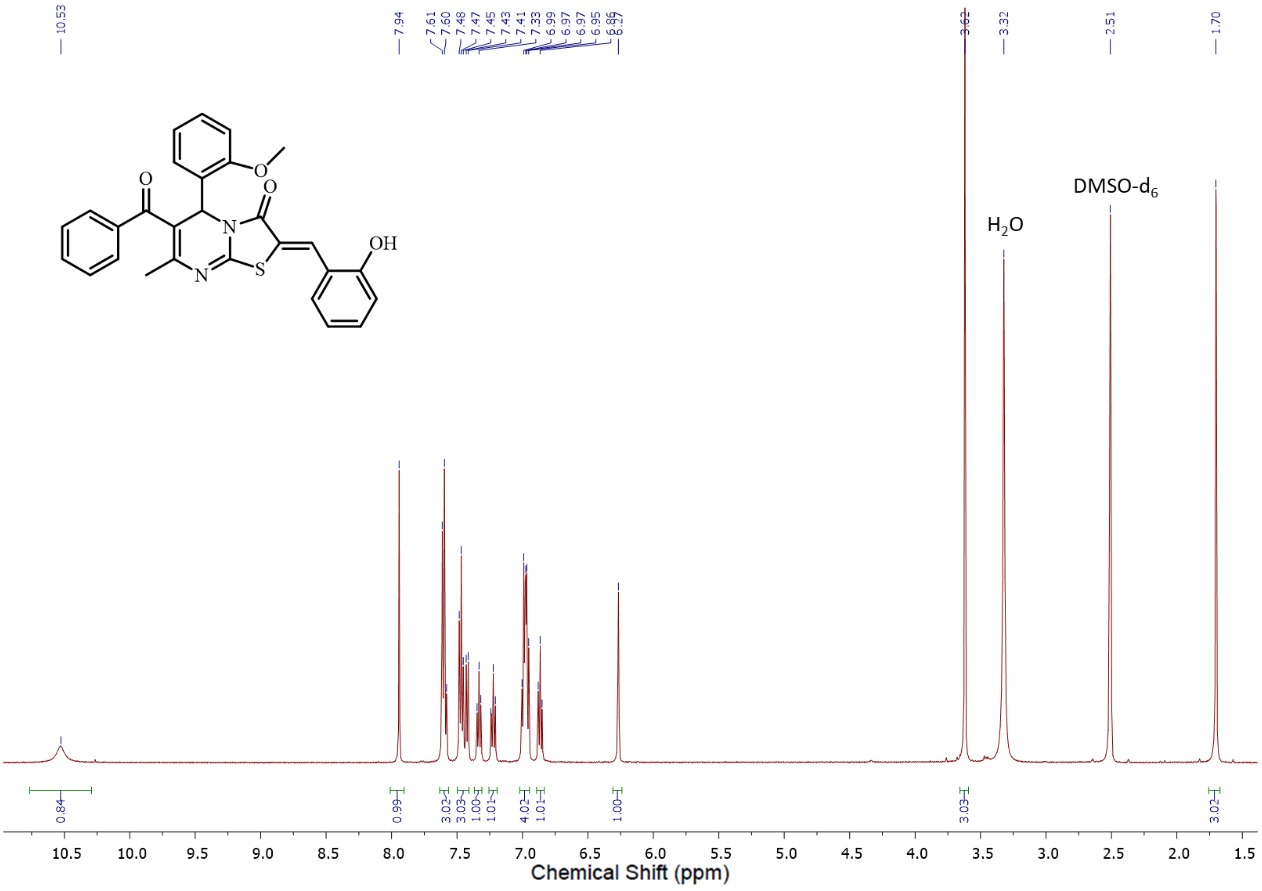

Figure S6. ^{13}C NMR spectrum of compound **11** ($DMSO-d_6$, 100 MHz, 25^{0}C).

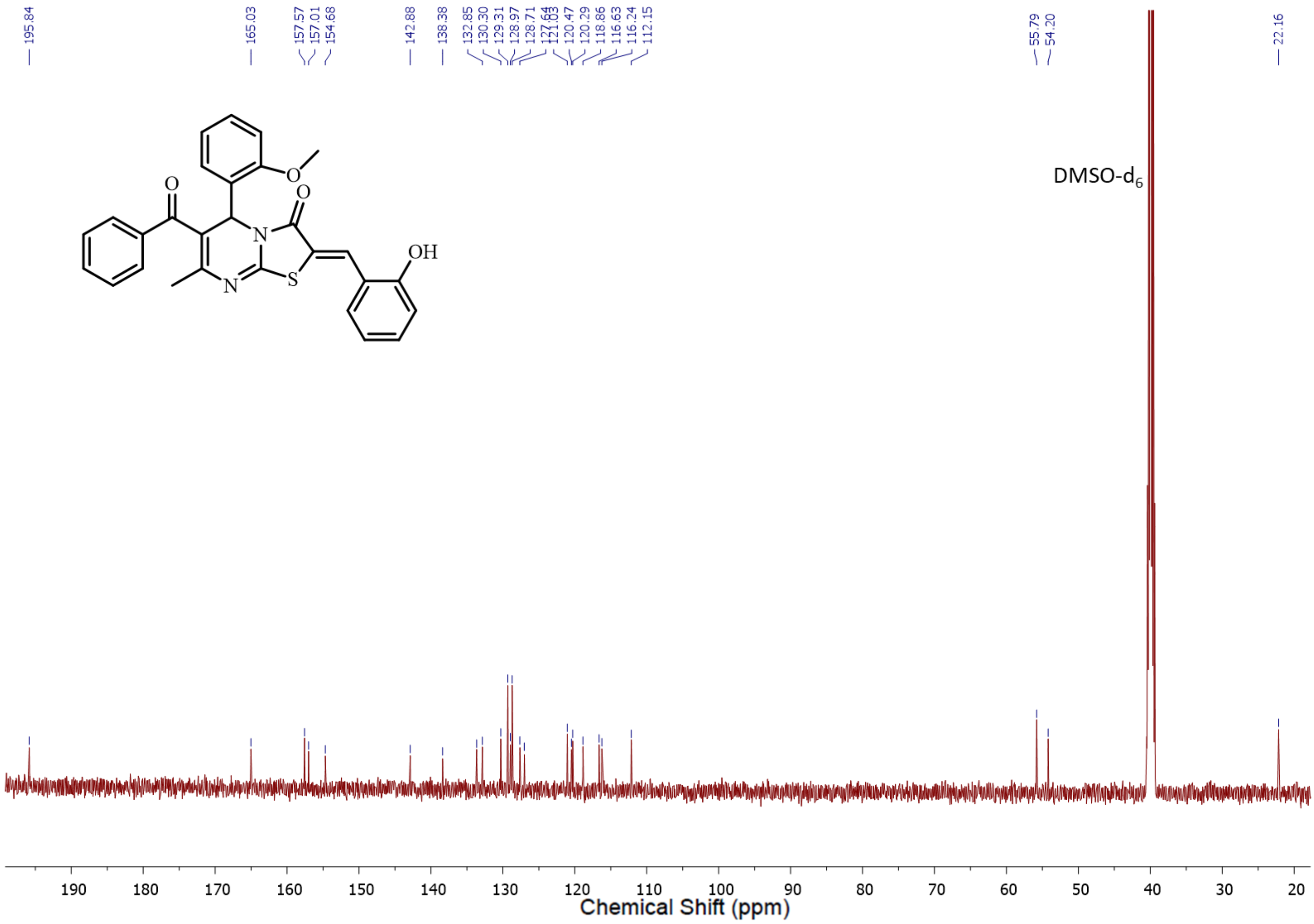

Figure S7. ESI MS spectrum of compound **11** (Ion Polarity: Positive).

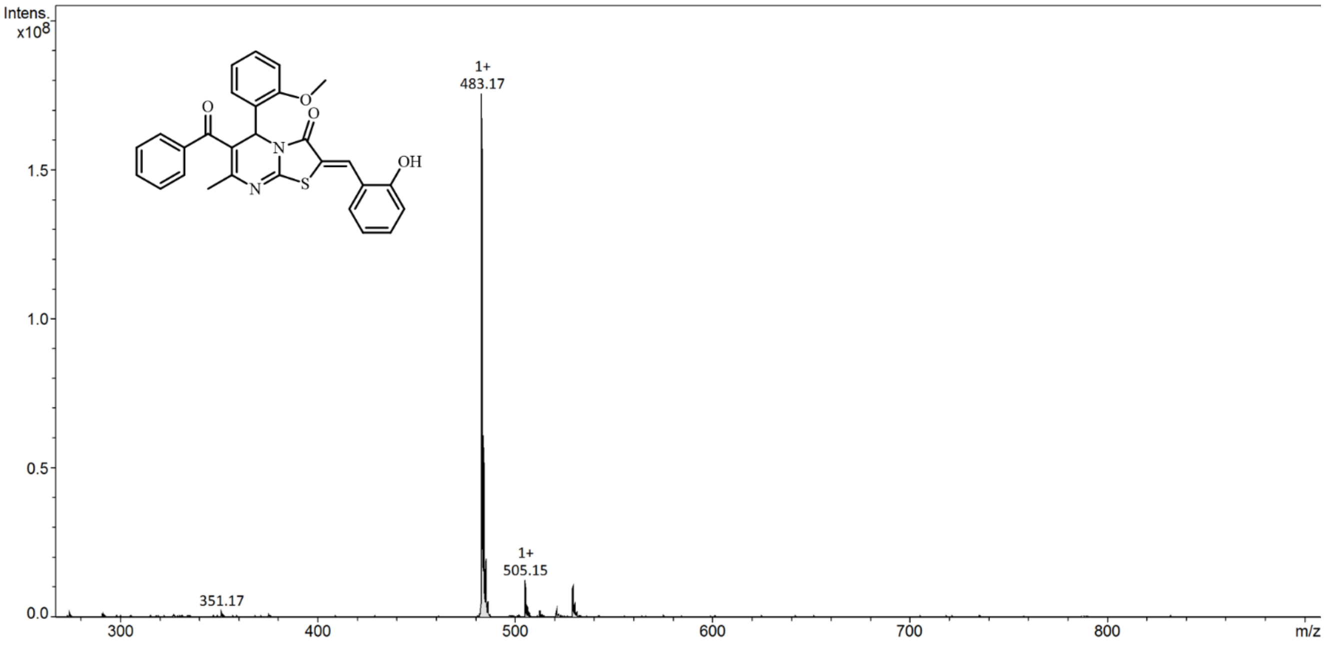

Figure S8. IR spectrum of compound **11** (KBr tablet).

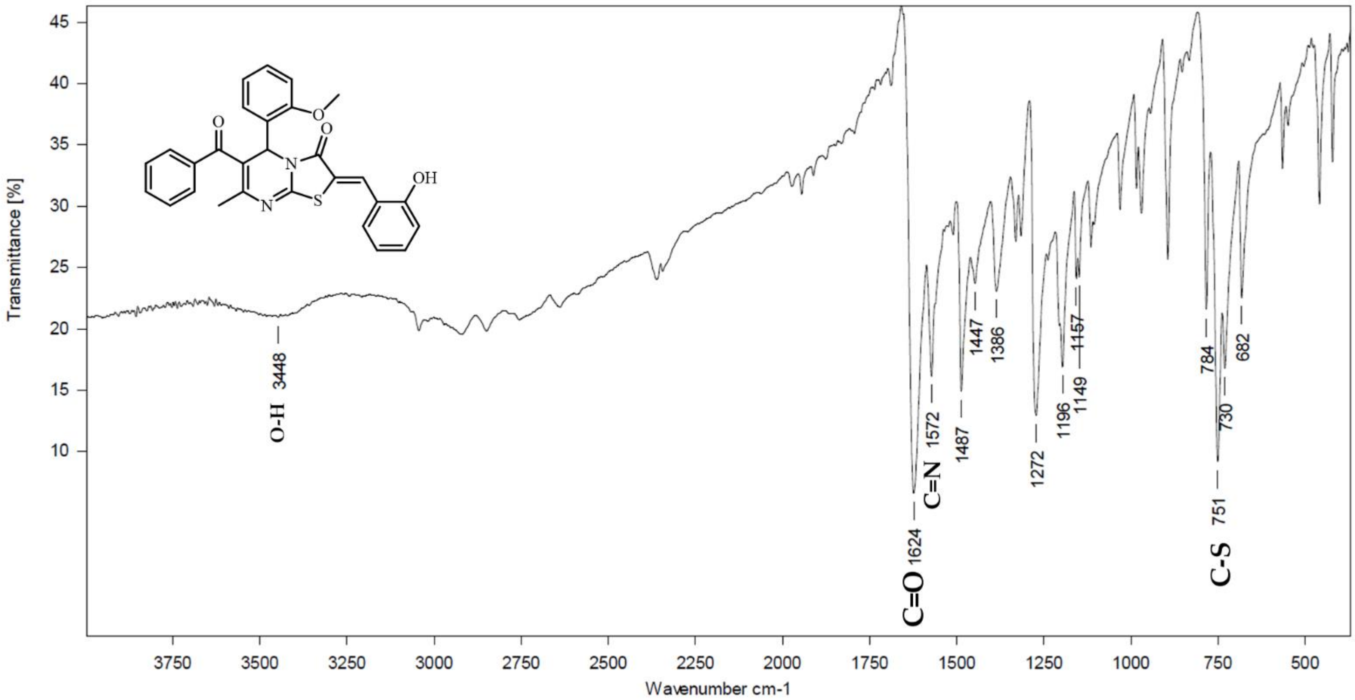

Figure S9. ^{1}H NMR spectrum of compound **12** (DMSO-d6, 400 MHz, 25[0]C).

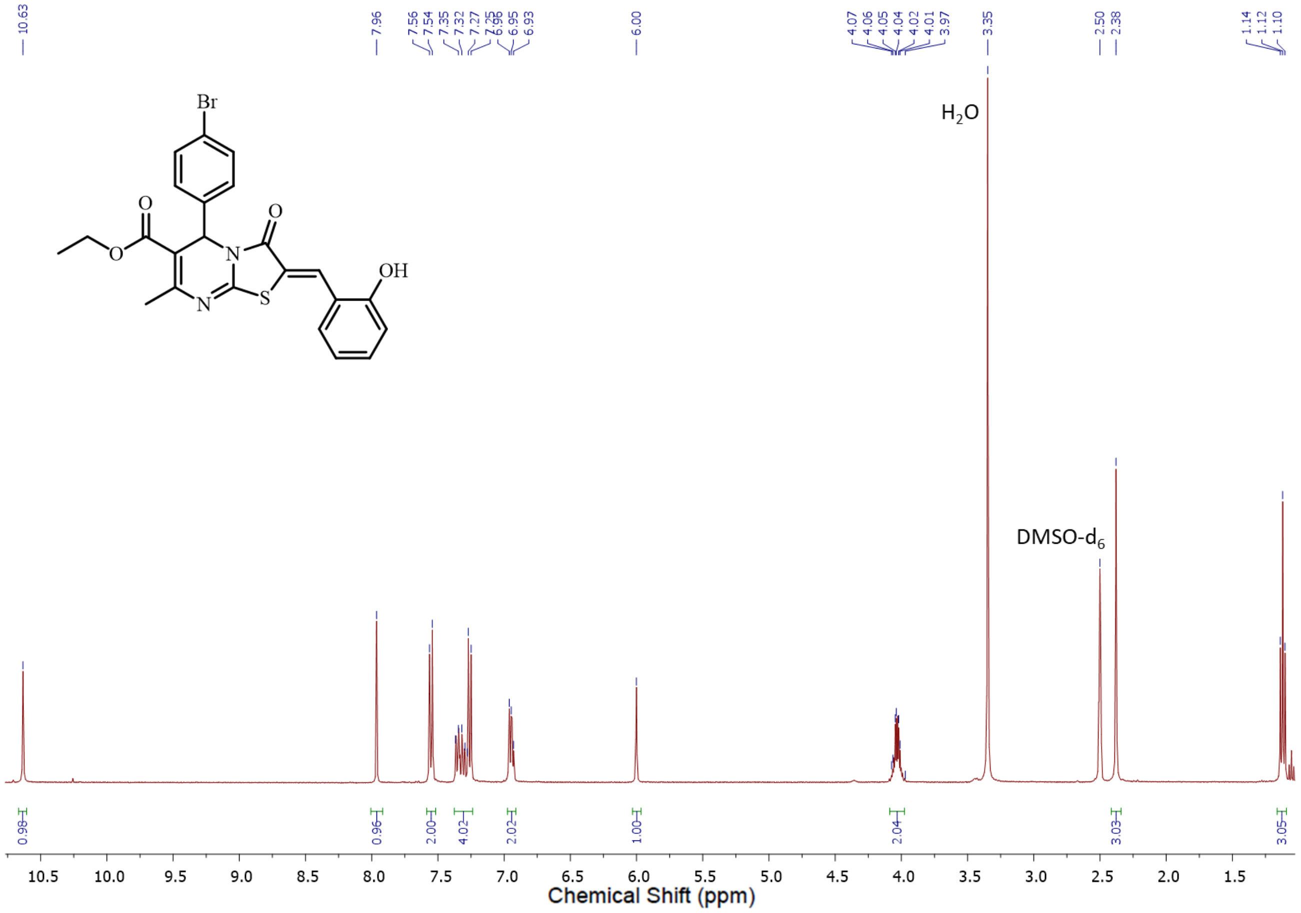

Figure S10. ^{13}C NMR spectrum of compound **12** (DMSO-d$_6$, 100 MHz, 25^{0}C).

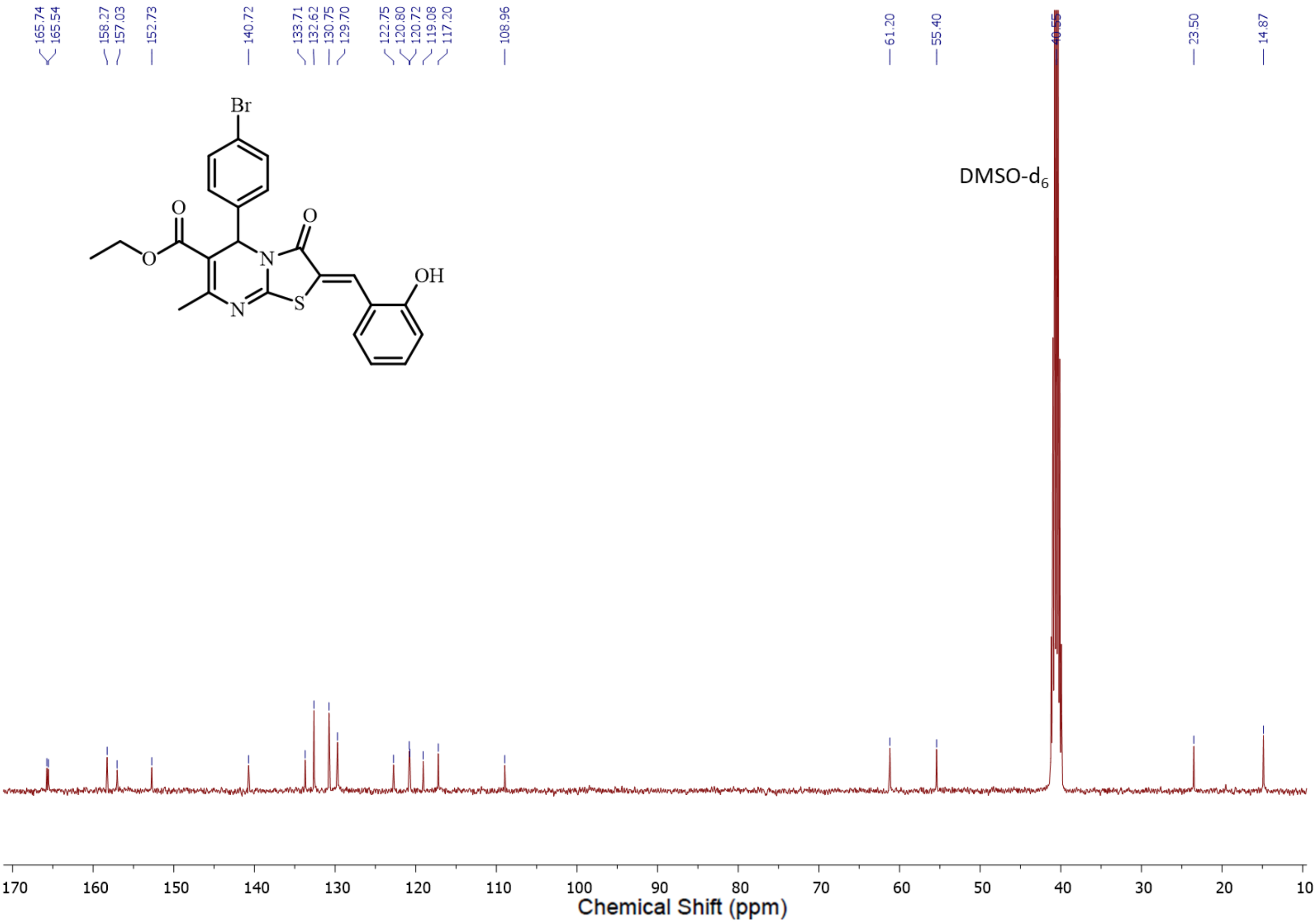

Figure S11. ESI MS spectrum of compound **12** (Ion Polarity: Positive).

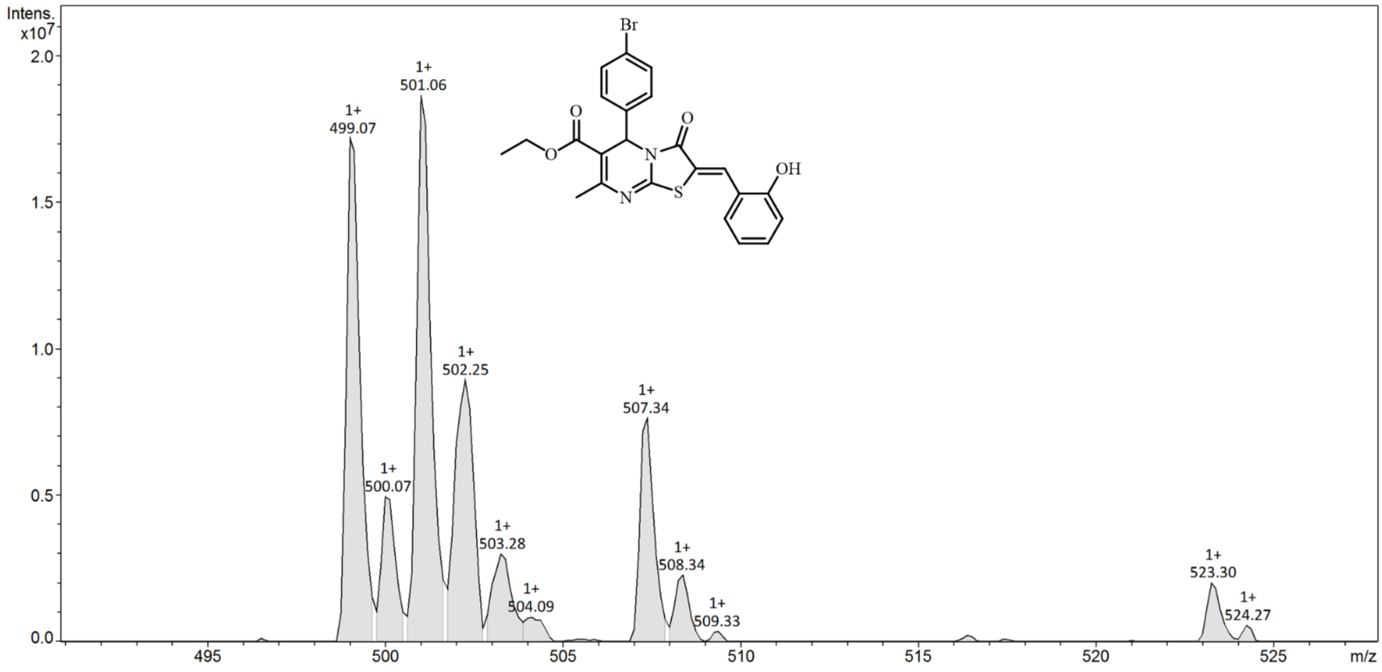

Figure S12. IR spectrum of compound **12** (KBr tablet).

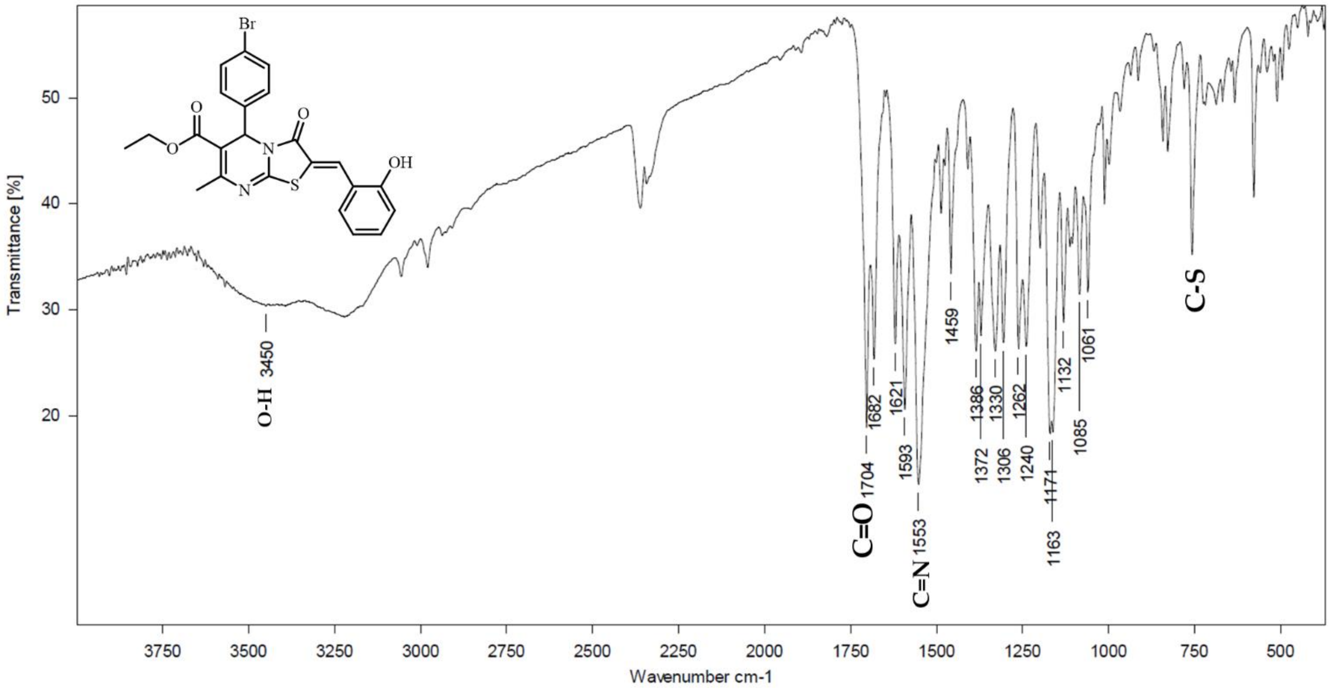

Figure S13. ^{1}H NMR spectrum of compound **14** (DMSO-d6, 600 MHz, 25[0]C).

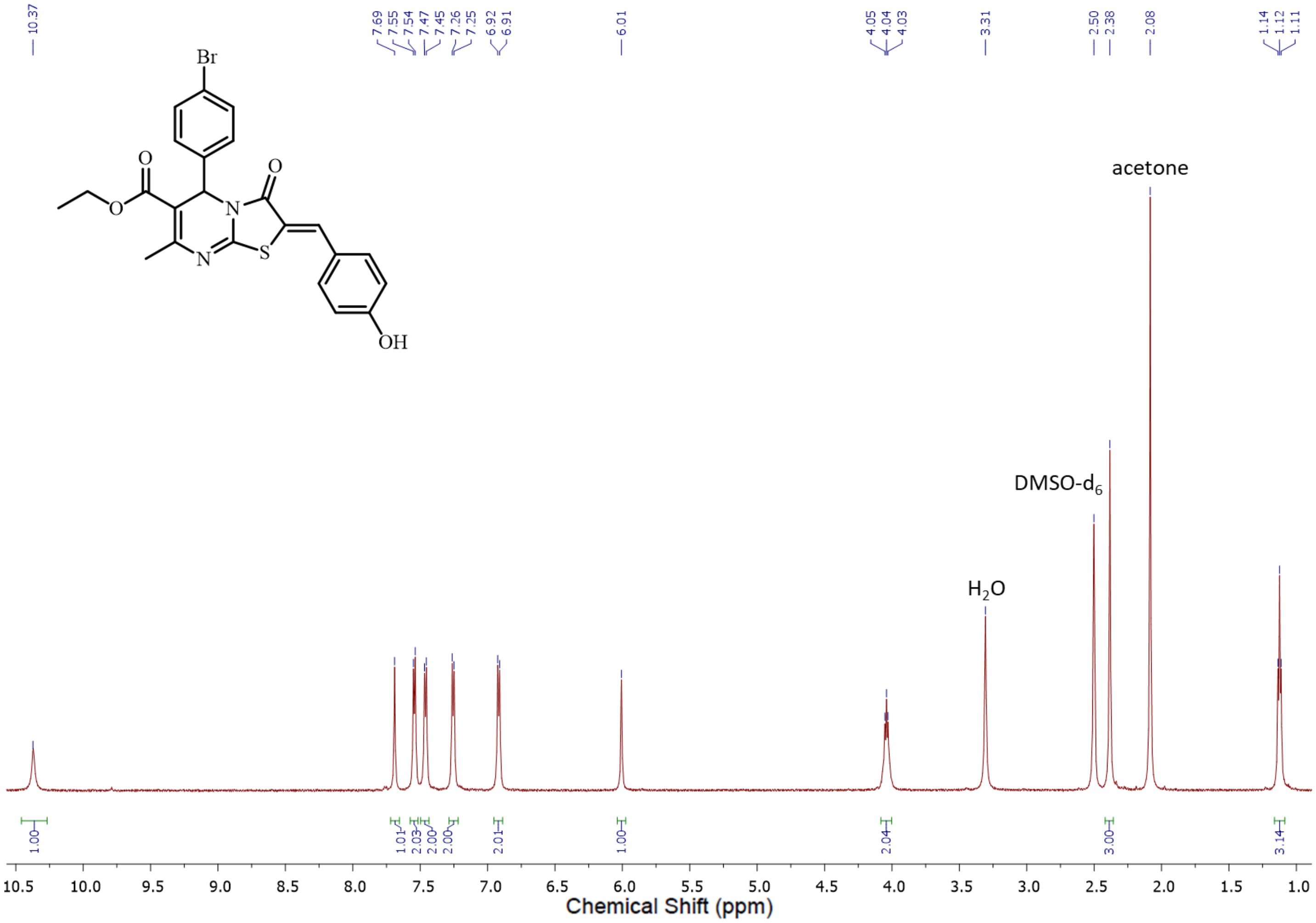

Figure S14. ^{13}C NMR spectrum of compound **14** (DMSO-d$_6$, 100 MHz, 25^{0}C).

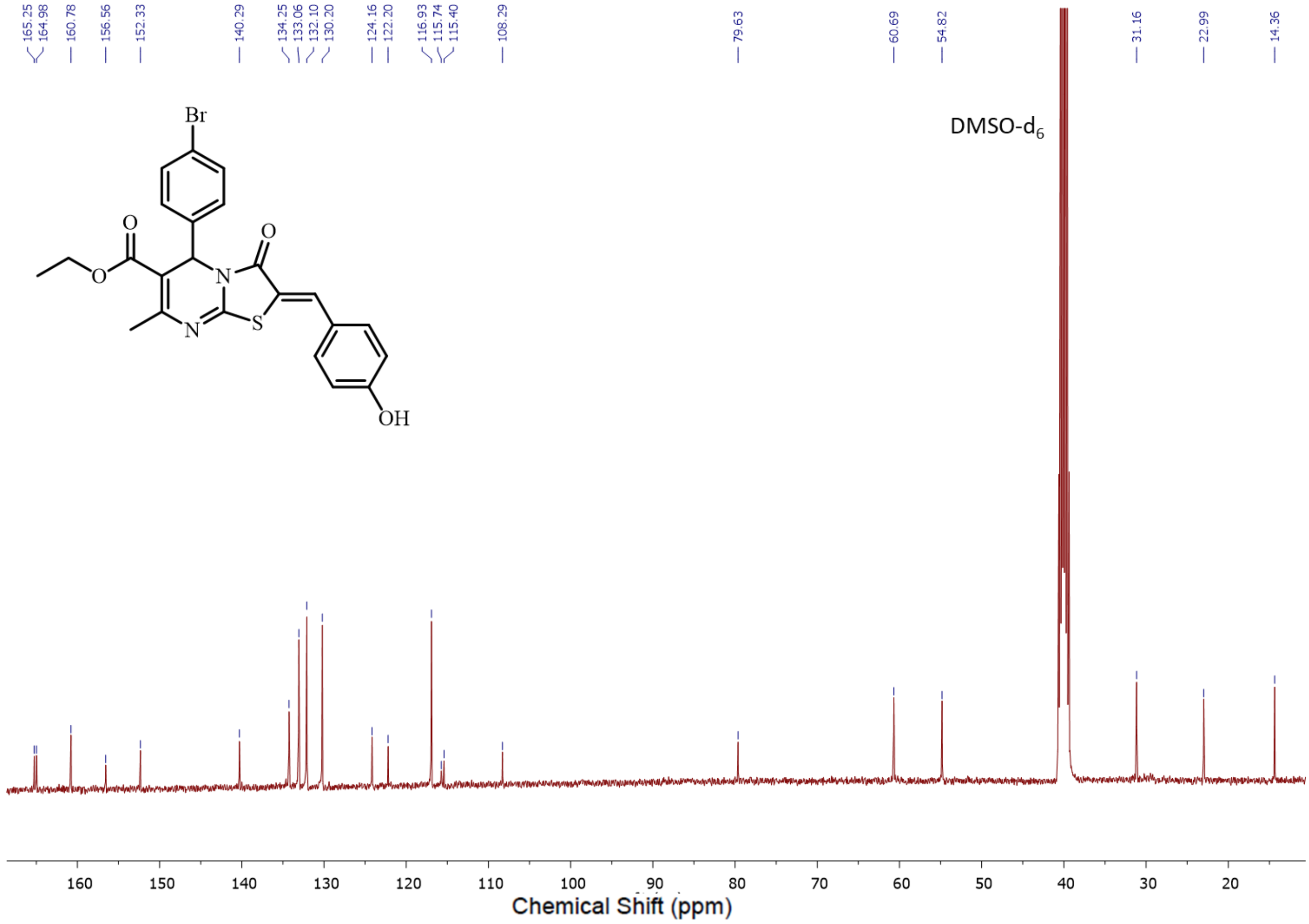

Figure S15. IR spectrum (KBr tablet) and ESI MS spectrum (Ion Polarity: Positive) of compound **14**.

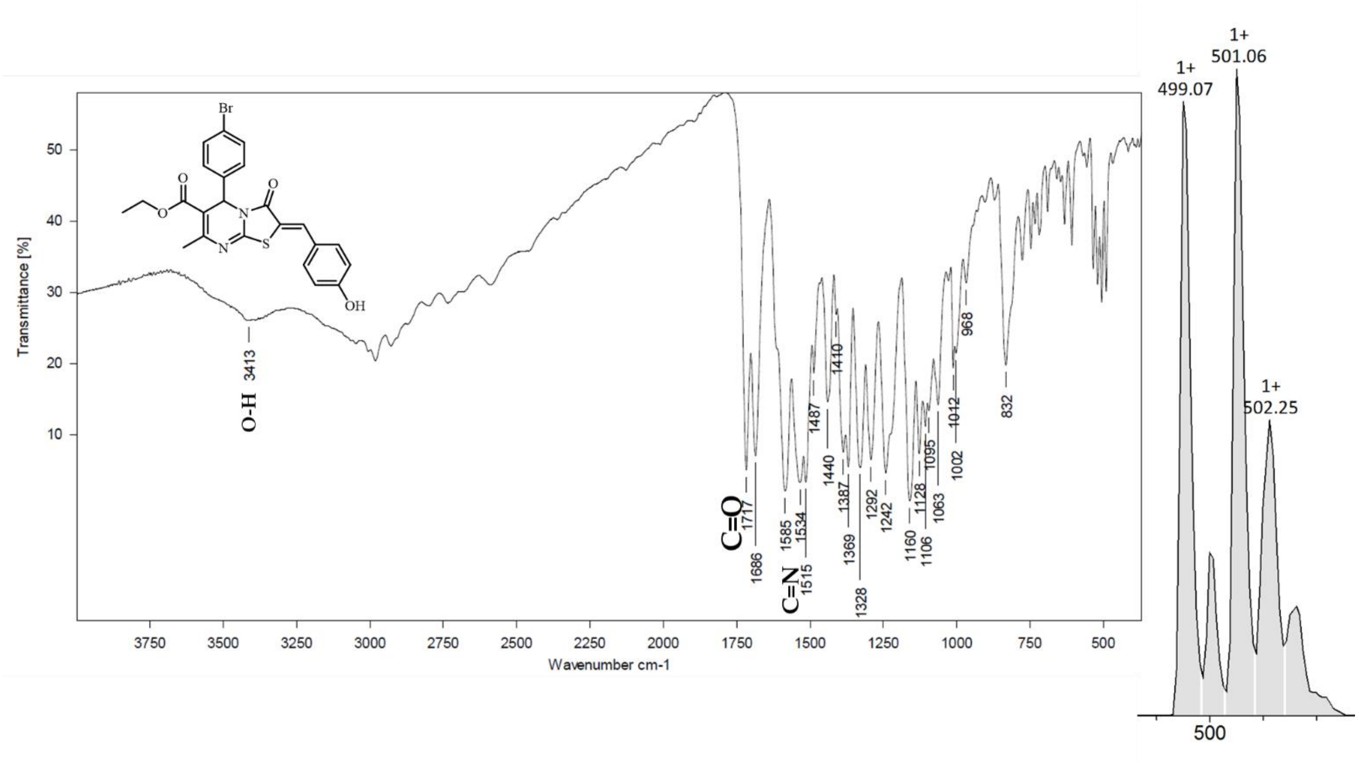

Figure S16. ^{1}H NMR spectrum of compound **15** (DMSO-d6, 500 MHz, 25^{0}C).

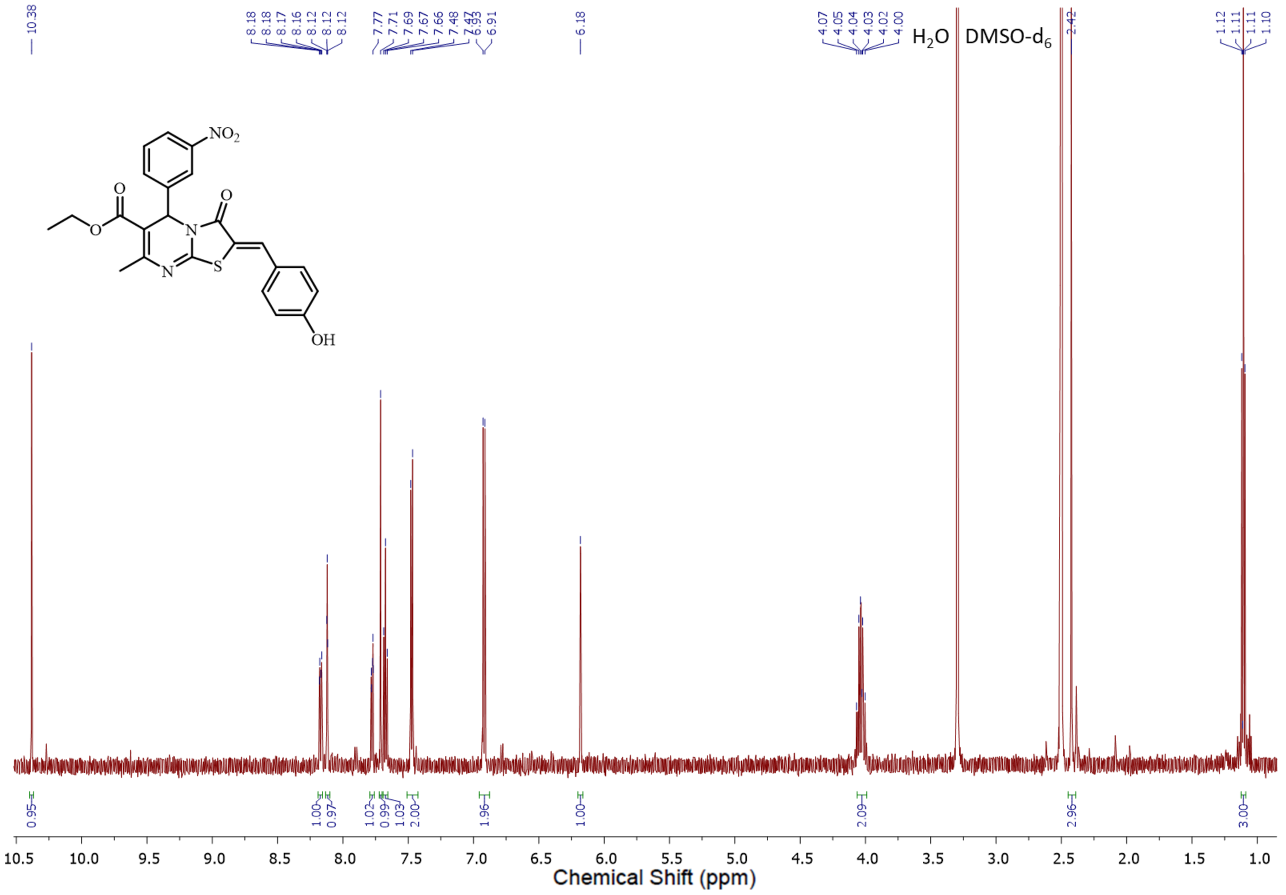

Figure S17. ^{13}C NMR spectrum of compound **15** (DMSO-d$_6$, 100 MHz, 25^{0}C).

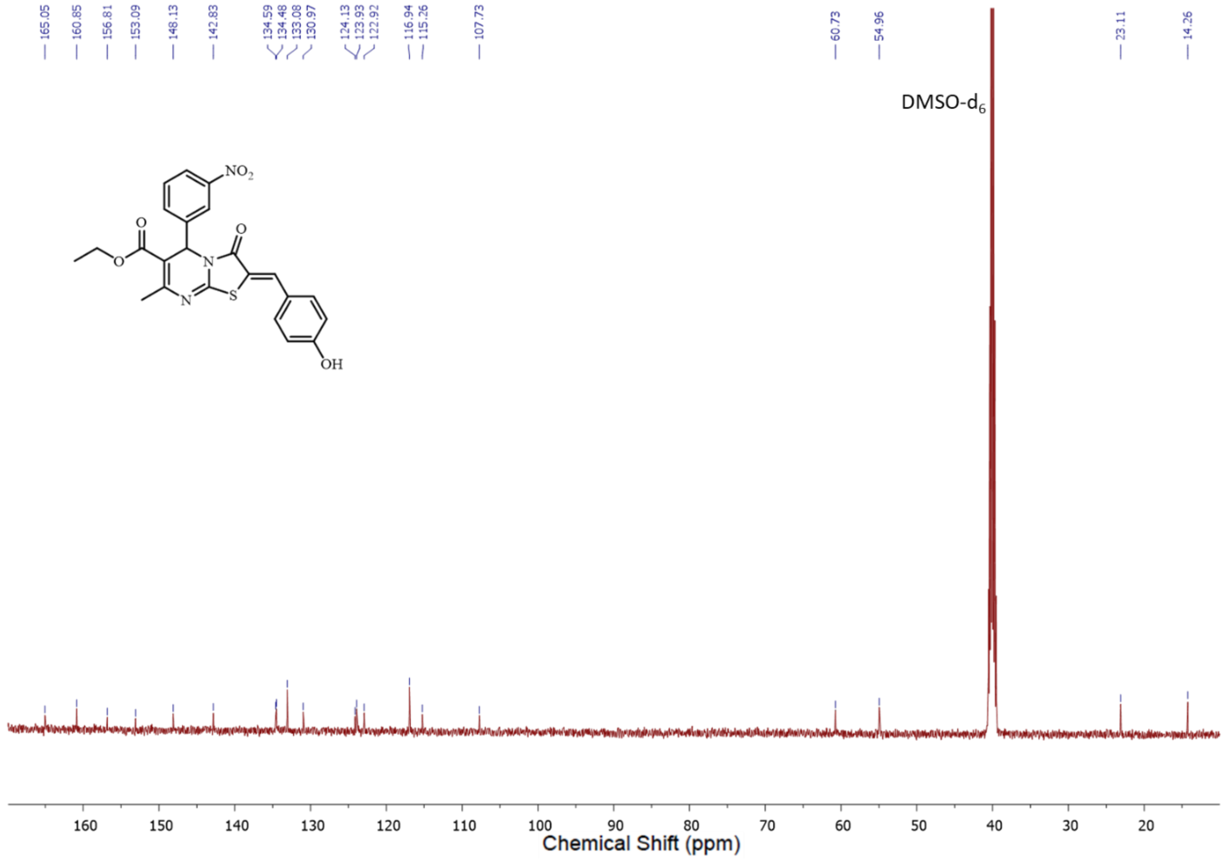

Figure S18. ESI MS spectrum of compound **15** (Ion Polarity: Positive).

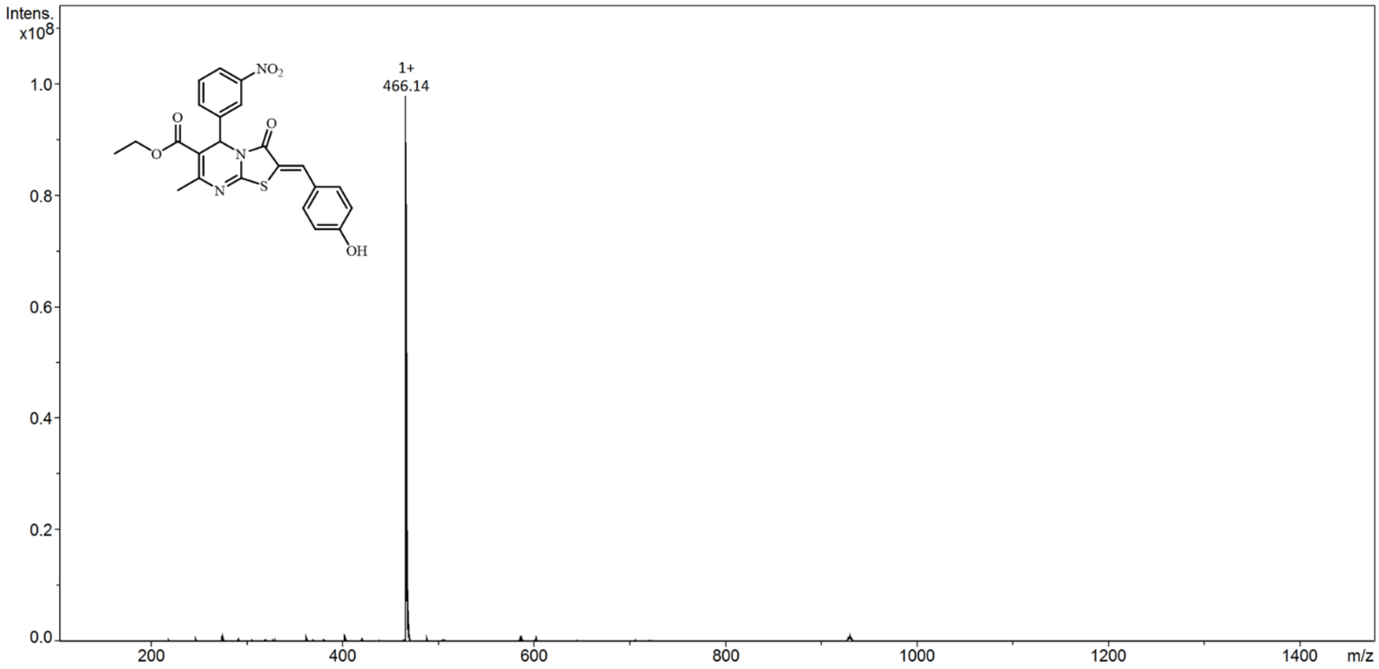

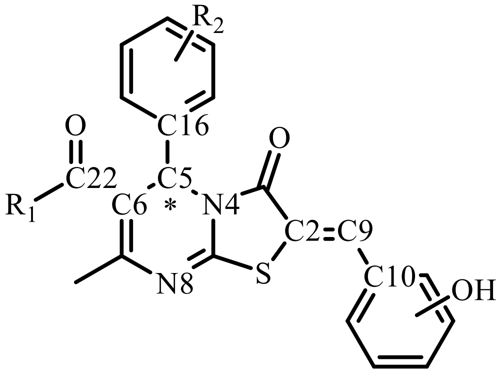

Table S1. Bond distances and angles of asymmetric C5 atom in studied compounds established by SCXRD.

Compound	**7**[1]	**8**[1]	**9**[1]	**9 DMSO**	**10**	**11**	**12**	**12-DMSO**	**13**	**14**	**14-DMSO**
d(C5-C6), Å	1.515(3)	1.508(2)	1.522(1)	1.518(1)	1.530(2)	1.522(2)	1.524(2)	1.516(2)	1.514(1)	1.522(3)	1.519(2)
d(N4-C5), Å	1.477(3)	1.481(2)	1.474(2)	1.475(1)	1.470(2)	1.478(1)	1.481(2)	1.475(1)	1.456(9)	1.474(3)	1.471(2)
d(N4-C3), Å	1.391(3)	1.395(2)	1.383(1)	1.390(1)	1.403(2)	1.371(2)	1.379(2)	1.389(2)	1.388(1)	1.395(2)	1.387(2)
d(C5-C16), Å	1.520(3)	1.533(2)	1.522(1)	1.518(1)	1.521(2)	1.520(1)	1.520(2)	1.524(2)	1.543(1)	1.518(3)	1.522(2)
∠C6-C5-N4, ^{0}	107.6(2)	109.34(2)	108.68(8)	107.65	108.3(1)	108.39(8)	108.5(1)	107.0(1)	108.3(6)	107.9(2)	108.2(6)
∠C6-C5-C16, ^{0}	113.3(2)	114.68(2)	110.05(8)	113.50(7)	110.9(1)	113.74(8)	112.7(1)	114.4(1)	113.0(7)	110.8(2)	112.9(6)
∠N4-C5-C16, ^{0}	109.4(2)	110.39(2)	110.91(8)	109.79(7)	110.2(1)	111.02(8)	110.2(1)	109.7(1)	108.9(7)	111.5(2)	109.9(6)

Table S2. Selected bond distances and dihedral angles for studied compounds established by SCXRD.

**Compound**	Dihedral angle between thiazolopyrimidine and the carbonyl group at C6 atom π-systems, ^{0}	Dihedral angle between thiazolopyrimidine and phenyl substituent of benzylidene fragment π-systems, ^{0}	d(C2-C9), Å	d(C9-C10), Å	d(C6-C22), Å
**7**[1]	13.41	2.40	1.345(3)	1.444(2)	1.486(2)
**8**[1]	9.96	14.82	1.348(2)	1.450(3)	1.475(3)
**9**[1]	14.12	6.24	1.350(1)	1.451(2)	1.485(2)
**9 DMSO**	15.09	2.36	1.349(1)	1.449(1)	1.478(1)
**10**	10.09	4.72	1.347(2)	1.450(2)	1.480(2)
**11**	39.13	9.00	1.350(2)	1.452(1)	1.497(1)
**12**	9.26	11.20	1.349(2)	1.450(2)	1.480(2)
**12-DMSO**	28.91	7.34	1.343(2)	1.453(2)	1.474(2)
**13**	7.15	4.81	1.367(2)	1.443(1)	1.472(1)
**14**	15.17	6.62	1.350(3)	1.450(3)	1.480(3)
**14-DMSO**	17.68	17.60	1.350(2)	1.446(2)	1.476(2)

Table S3. H- bond distances presenting in crystals of studied compounds.

**Compound**	**7-EtOH**[1]	**8**[1]	**9**[1]	**9 DMSO**[1]	**10-MeOH**	**11**	**12**	**12-DMSO**	**13**	**14**	**14-DMSO**
d(O3-O11), Å	-	-	2.696(1)	-	-	2.672(1)	2.651(2)	-	-	-	-
d(O11-O_{DMSO}), Å	-	-	-	2.664(1)	-	-	-	2.624(1)	-	-	2.612(2)
d(O13-N8), Å	-	2.687(1)	-		-	-	-	-	2.725(9)	2.797(2)	-
d(O11-$O_{EtOH/MeOH}$), Å	2.639(3)	-	-	-	2,712(1)	-	-	-	-	-	-
∠C11-O11-O3, ^{0}	-	-	121.64(7)	-	-	120.54(7)	120.7(1)	-	-	-	-
∠C11-O11-O_{DMSO}, ^{0}	-	-	-	112.40(6)	-	-	-	112.16(8)	-	-	-
∠C13-O13-O_{DMSO}, ^{0}	-	-	-	-	-	-	-	-	-	-	116.1(1)
∠C11-O11-$O_{EtOH/MeOH}$, ^{0}	118.0(1)	-	109.99(8)	-	-	-	-	-	-	-	-
∠C11-O11-N8, ^{0}	-	112.47(1)	-	-	-	-	-	-	-	-	-
∠C13-O13-N8, ^{0}	-	-	-	-	-	-	-	-	120.3(5)	116.1(1)	-

Figure S19. Fragment of crystal packing of **11-DMSO** showing the intermolecular H-, Br/π-, and π-π bonding (red, brown and blue dotted lines, respectively).

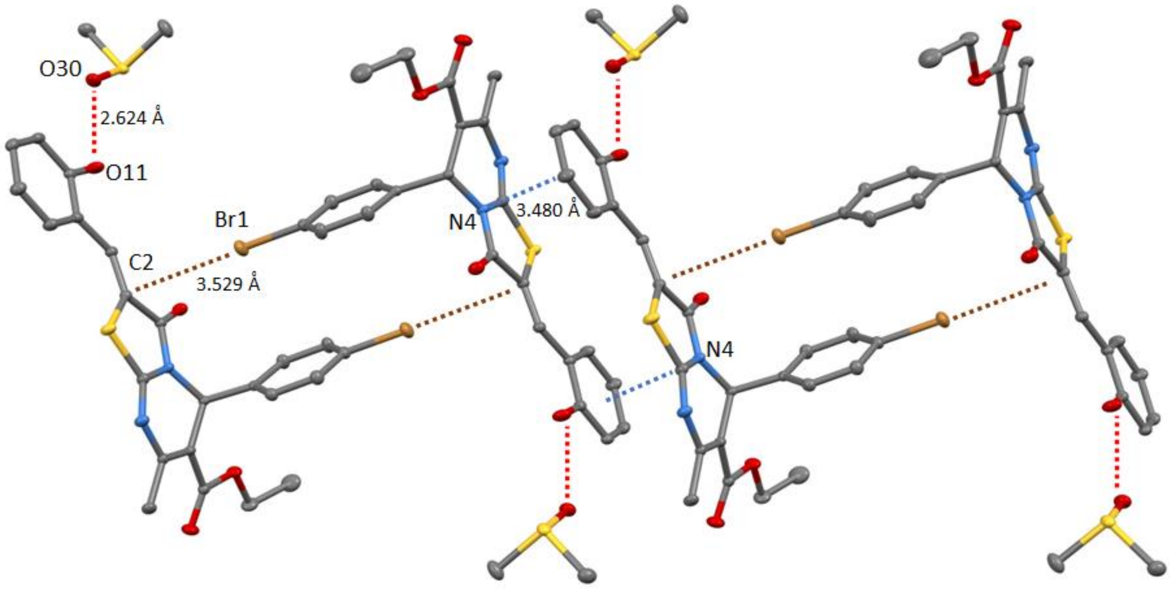

Figure S20. Fragment of crystal packing of **14** showing the intermolecular H-, Br/π-, and π-π bonding (red, brown and blue dotted lines, respectively).

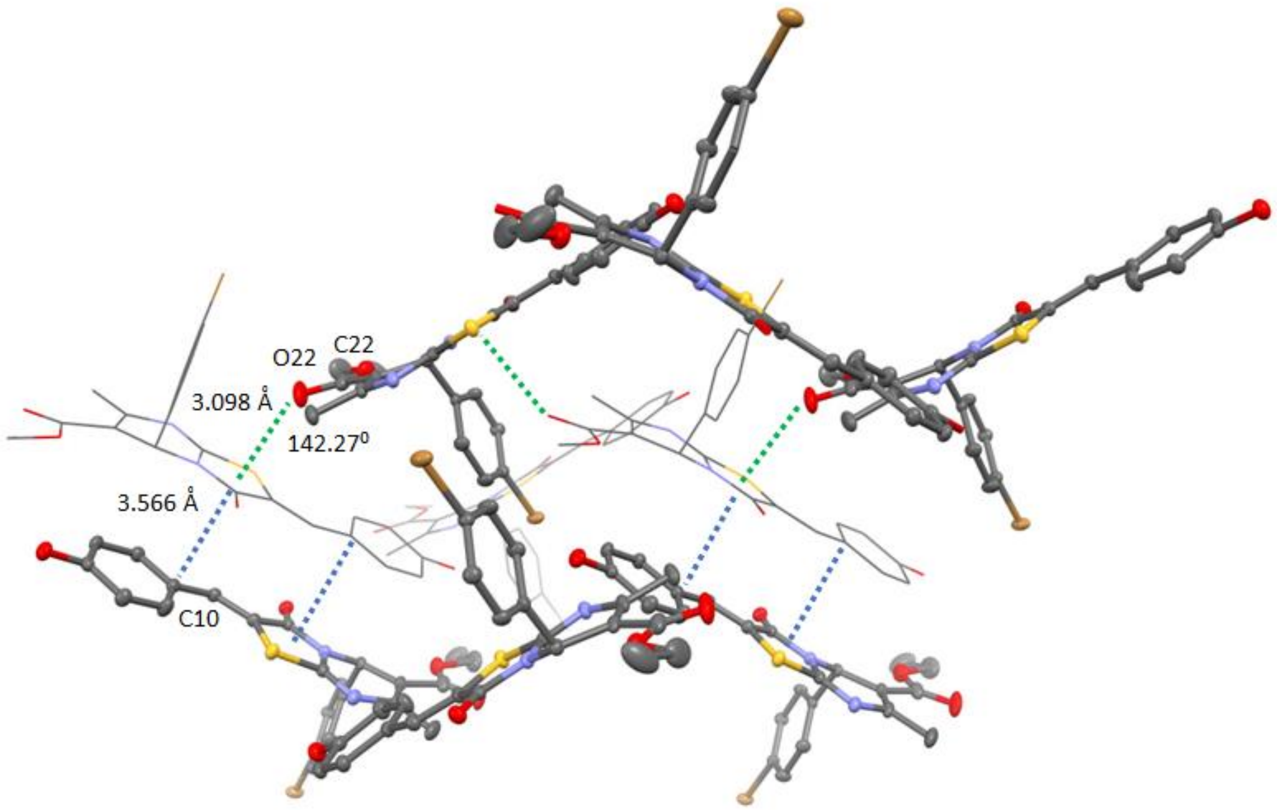

Figure S21. Fragment of crystal packing of **14-DMSO** showing the intermolecular H-, Br/π-, and π-π bonding (red, brown and blue dotted lines, respectively).

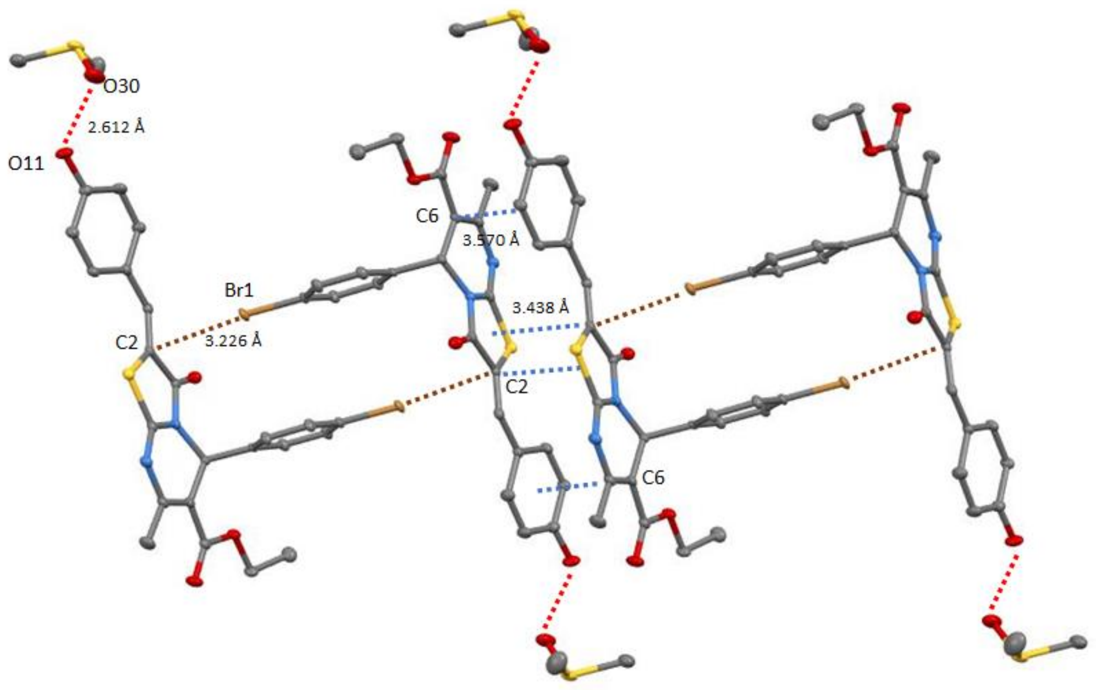

Supplement: Supplementary file 1 [file molecules-27-07747-s001.zip › molecules-2002209-supplementary.pdf]
